# Recruitment of dorsal midbrain catecholaminergic pathways in the recovery from nerve injury evoked disabilities

**DOI:** 10.1186/s12990-015-0049-7

**Published:** 2015-08-18

**Authors:** David Mor, James W M Kang, Peter Wyllie, Vignaraja Thirunavukarasu, Hayden Houlton, Paul J Austin, Kevin A Keay

**Affiliations:** School of Medical Sciences, Discipline of Biomedical Sciences, The University of Sydney, C42, Cumberland Campus, Lidcombe, NSW 2141 Australia; School of Medical Sciences, Discipline of Anatomy and Histology, The University of Sydney, Sydney, NSW 2006 Australia

**Keywords:** Periaqueductal gray, Sciatic nerve, Injury, Pain, Social behavior, Dopamine, Dorsal raphe

## Abstract

**Background:**

The periaqueductal gray region (PAG) is one of several brain areas identified to be vulnerable to structural and functional change following peripheral nerve injury. Sciatic nerve constriction injury (CCI) triggers neuropathic pain and three distinct profiles of changes in complex behaviours, which include altered social and sleep–wake behaviours as well as changes in endocrine function. The PAG encompasses subgroups of the A10 dopaminergic and A6 noradrenergic cell groups; the origins of significant ascending projections to hypothalamic and forebrain regions, which regulate sleep, complex behaviours and endocrine function. We used RT-PCR, western blots and immunohistochemistry for tyrosine hydroxylase to determine whether (1) tyrosine hydroxylase increased in the A10/A6 cells and/or; (2) de novo synthesis of tyrosine hydroxylase, in a ‘*TH*-*naïve*’ population of ventral PAG neurons characterized rats with distinct patterns of behavioural and endocrine change co-morbid with CCI evoked-pain.

**Results:**

Evidence for increased tyrosine hydroxylase transcription and translation in the constitutive A10/A6 cells was found in the midbrain of rats that showed an initial 2–3 day post-CCI, behavioural and endocrine change, which recovered by days 5–6 post-CCI. Furthermore these rats showed significant increases in the density of TH-IR fibres in the vPAG.

**Conclusions:**

Our data provide evidence for: (1) potential increases in dopamine and noradrenaline synthesis in vPAG cells; and (2) increased catecholaminergic drive on vPAG neurons in rats in which transient changes in social behavior are seen following CCI. The data suggests a role for dopaminergic and noradrenergic outputs, and catecholaminergic inputs of the vPAG in the expression of one of the profiles of behavioural and endocrine change triggered by nerve injury.

## Background

The relationships of injury, the sensation of pain and the affective-motivational pain state are not straightforward, because they are not wholly consequent upon each other. Nerve damage due to trauma, disease or surgical intervention does not always trigger a neuropathic pain state in humans [1]. There is also no simple relationship between pain intensity and reported disruptions to quality of life [2]. Further, there exists a, ‘*non*-*patient* chronic pain population’ that reports sensory dysfunction, but does not seek treatment and thus is ‘clinically invisible’. The patient population that does seek medical assistance for chronic pain conditions invariably presents with more than just sensory dysfunction. Their pain state in many cases includes problems with sleep [3–7] and impaired social functioning [7–9].

Problems with sleep, defined by measuring disrupted sleep–wake cycles, are also triggered by nerve injury in rat models of neuropathic pain (although c.f. [10]). Spared nerve injury increases the frequency of episodes of wakefulness and slow-wave sleep [11]. Similarly, chronic constriction injury of the sciatic nerve (CCI) also disrupts the sleep–wake cycle, the nature of these disruptions depends upon the strain of the rat, the diurnal phase of measurement, the housing conditions, the post-injury time of recordings and whether one or two nerves are ligated [12–14]. A unilateral CCI triggers increased wakefulness and decreased sleep from days 2 to days 10 post-injury [12], a similar pattern is also reported following bilateral CCI at 7 days post injury [13]. We have also reported that CCI triggers sleep-wake cycle disturbances during the first week post-injury, and further we have reported that the patterns of these changes differ in distinct subgroups of rats. The sleep–wake cycle changes that we have reported correspond directly with changes in resident-intruder behaviours in a social-interactions test [14]. The sleep–wake cycle and behavioural changes were not driven by differing levels of sensory dysfunction as all rats showed identical degrees of increased sensitivity to mechanical and cold stimuli [14, 15]. The fact that sleep–wake cycle changes occurred only in animals whose resident intruder behaviour also changed corresponds with data in human populations showing a correlation between sleep disturbances and social dysfunction(s) in neuropathic pain patients [3–7].

In detail, our data showed that CCI had no effect on sleep-wake cycles in approximately half of the rats given a CCI. Neither did these rats show changes in behaviour in the resident-intruder test [14, 15]. This group of rats was classified as having *Pain alone*. A second subgroup, of ~30% of the rats, showed reduced slow-wave sleep (SWS) and increased wakefulness during both the dark and light phases, as well as reductions in dominance behaviours and increased non-social behaviours in the resident-intruder test. These rats were classified as *Pain andDisability* rats [14, 15]. The remaining CCI rats (~20%) showed reduced SWS and increased wakefulness during the light phase only, and a transient (2–3 day) reduction in dominance behaviour and increased non-social behaviour in the resident-intruder test, these rats were classified as *Pain and Transient Disability* rats [14, 15].

It has been shown that the duration and frequency of periods of wakefulness are regulated by dopamine (DA) containing neurons located in the ventral half of the periaqueductal gray (PAG) [16]. This region encompasses the ventrolateral column of the periaqueductal gray (vlPAG) and the dorsal raphe nucleus (DRN). Increased activity of these DA containing neurons is suggested to regulate the activity of a specific population of hypothalamic neurons, which prevent the switch between wakefulness and sleep states [16]. Furthermore, noradrenergic neurons in this same region that form part of the rostral extension of the A6 cell group [17] have also been shown to promote wakefulness in the rat [18]. In rats, evidence of significant impact of nerve injury on the PAG has been revealed in a number of studies, and it appears to be one of several CNS sites particularly vulnerable to the effects of peripheral nerve injury [19–21]. This vulnerability may be due in part to the substantial direct and somatotopically ordered inputs from peripheral nerve recipient regions of the spinal cord and brainstem [22]. It is possible therefore, that the different patterns of sleep–wake cycle changes observed in the *Pain and Disability* and *Pain and Transient Disability* rats after sciatic nerve CCI may be contributed to by: (1) selective increases in the activity of dopaminergic and or noradrenergic cells located in the ventral half of the PAG and/or; (2) de novo synthesis of dopamine, or noradrenaline, in a ‘*dopamine*-*naïve*’ and “*noradrenaline*-*naïve*” populations of ventral PAG neurons.

The PAG encompasses the group of DA neurons, referred to as the dorso-caudal extension of the A10 group (A10dc). This group includes neurons of the DRN, the vlPAG and also cells in the lateral column of the periaqueductal gray (lPAG) [23–26]. A majority of the A10dc cell group neurons are found in the DRN, where they are co-located with serotonergic neurons. However, they share similar projection targets and electrophysiological properties to the A10 cells of the ventral tegmental area (VTA) [25, 27]. The PAG also encompasses, a small group of noradrenergic neurons described as the rostral extension of the A6 cell group [17].

Using RT-PCR and western blots, relative levels of transcription and translation of tyrosine hydroxylase were quantified in the ventral PAG, A10dc/A6 [rostral] cell group region, to determine the effects of CCI 6 days after injury, in rats with *Pain alone*, *Pain and Disability* and *Pain and Transient Disability*. The differences between these three behavioural sub-groups were then investigated using immunohistochemistry to determine the location/s of TH protein expression changes revealed using western blot analyses.

## Results

### Resident-intruder testing

**Fig. 1:**
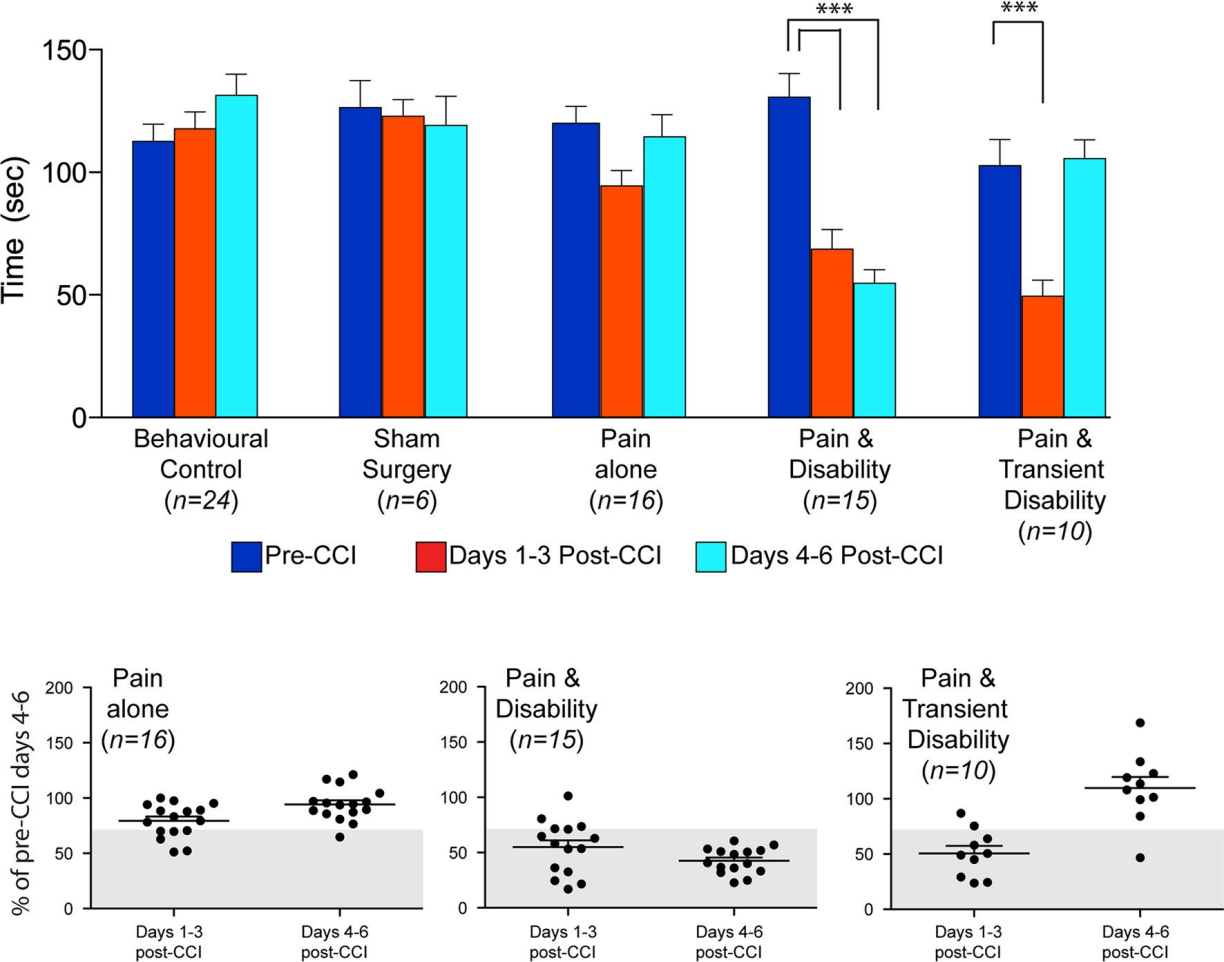
Levels of dominance behaviour before and after nerve injury. *Top panel* mean durations (in seconds) of dominance behaviour (±SEM) measured: pre-injury (days 3–5); days 1–3 post-injury and; days 4–6 post-injury in behavioural controls, sham surgery rats and rats with *Pain alone*, *Pain and Disability* and *Pain and Transient Disability*. Significance with respect to pre-injury days is shown ***p < 0.001 (ANOVA, and post hoc Fischer’s *PLSD*). *Lower panel* individual data for *Pain alone*, *Pain and Disability* and *Pain and Transient Disability* post-CCI behavioural groups. Data are expressed as the mean percentage change from pre-CCI levels for days 1–3 post-CCI and days 4–6 post-CCI. The shaded area indicates a 30% reduction from pre-injury dominance behaviours. Rats that showed no differences in their post-CCI dominance behaviour were defined as *Pain alone* rats. Rats with a decrease of at least 30% in the duration of their dominance behaviours on 4 or more of the 6 days post injury days were defined as *Pain and Disability* rats. The rats that showed a 30% or more reduction in their dominance behaviours for days 1–3 post-CCI but then returned to pre-CCI levels were considered *Pain and Transient Disability* rats.

Replicating our previous findings, rats could be categorised into three groups post-CCI based upon changes in their dominance and non-social behaviours in the resident-intruder, social interactions test [14, 19–21]. Of the 55 rats that underwent CCI and daily resident-intruder testing, fifty-four percent (30/55) were classified as *Pain alone*, as their dominance behaviours in response to the presence of an intruder did not decrease significantly following nerve injury. Twenty-seven percent (15/55) showed persistent reductions in dominance levels following the injury and were classified as *Pain and Disability* rats. Nineteen percent (10/55) rats showed a transient reduction in dominance levels in the first 2–3 days following injury but returned to pre-injury levels during days 4–6 and were classified as *Pain andTransient Disability* rats. These findings are summarised in Fig. 1.

There were no differences in the levels of dominance during the first 5 days of resident-intruder testing in any of the behavioural or control groups (F_14,198_ = 9.27, NS). The dominance levels in behavioural and sham controls did not differ throughout the eleven, day testing period (see Fig. 1). *Pain and Disability* rats showed a reduction in dominance levels on both days 1–3 and 4–6 post-injury in comparison to their pre-injury levels (F_14,198_ = 9.27, p < 0.001), as well as compared with dominance levels throughout testing in each of the control groups (F_14,198_ = 9.27, p < 0.001). *Pain and Transient Disability* rats showed a significant reduction in dominance levels on days 1–3 post-injury compared with pre-injury levels (F_14,198_ = 9.27, p < 0.05), as well as each of the control groups (F_14,198_ = 9.27, p < 0.001 vs, behavioural controls and p < 0.01 vs, sham controls), dominance levels returned to pre-injury levels on days 4–6 post CCI. The slight, but non-significant reduction in dominance seen in *Pain alone* rats was attributable solely to a small reduction on the day following surgery.

### Real-time RT-PCR and Western blotting

**Fig. 2:**
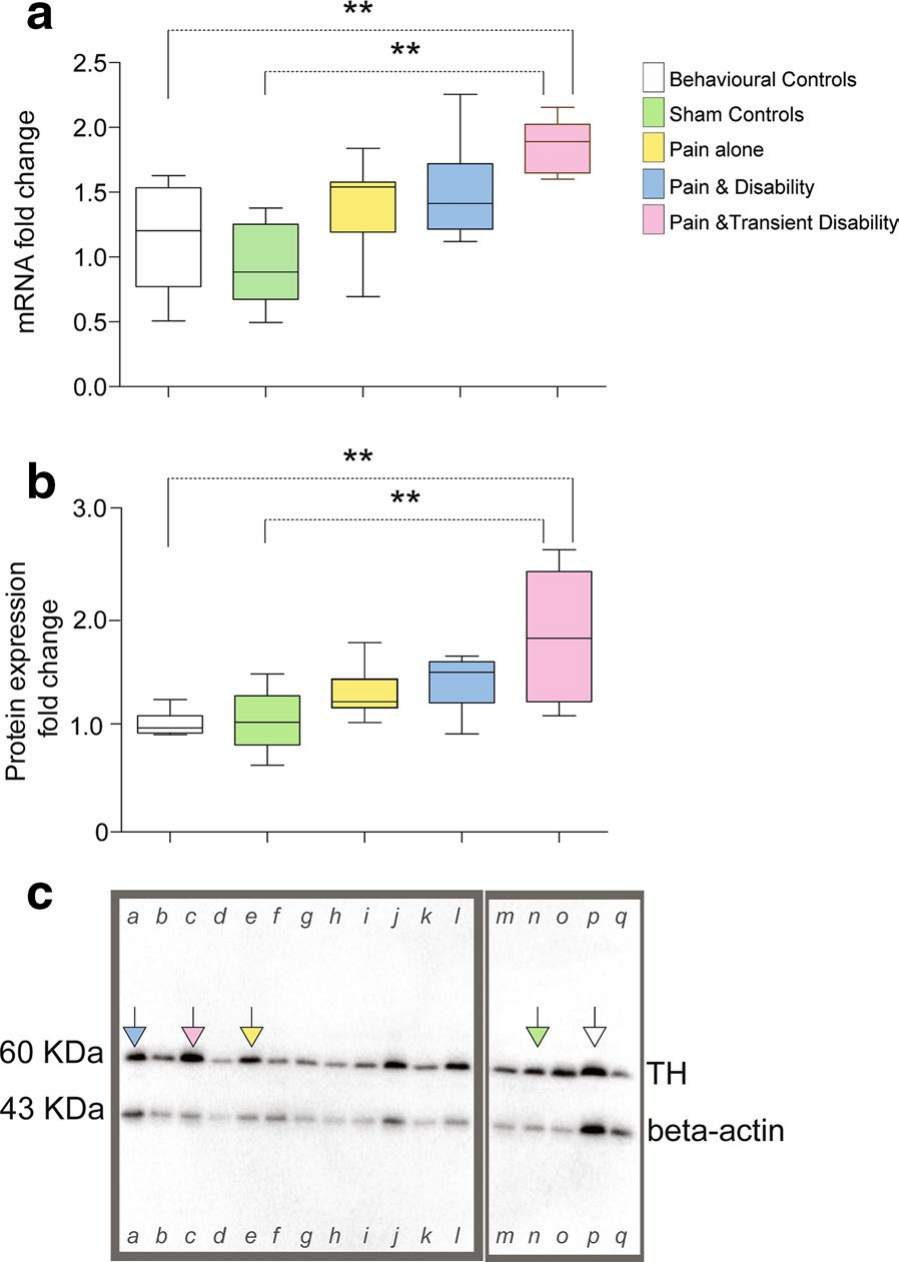
TH mRNA and protein expression in rats with distinct patterns of injury-triggered disability. *Box* and *whisker plots* showing: **a** relative TH mRNA expression and; **b** relative TH protein expression as determined by real-time RT-PCR and western blotting, respectively. Data are shown for behavioural controls (*white*); sham controls (*green*); *Pain alone* (*yellow*); *Pain and Disability* (*blue*); and *Pain and Transient Disability* (*pink*). Significance with respect to behavioural controls and sham surgery controls are shown **p < 0.01 (one-way ANOVA, post hoc Bonferroni test). **c** Examples of two different western blots showing fluorescence for TH protein (60 KDa) and beta-actin (43 KDa). In each example gel specific examples of a protein sample from each control and experimental group, are indicated by a *different coloured arrow*: behavioural controls (*white*); sham controls (*green*); *Pain alone* (yellow); *Pain and Disability* (*blue*); and *Pain and Transient Disability* (*pink*). (We have shown entire gels in this figure, *Pain alone* rats in *lanes* *e*, *f*, *g*, *h*, *I*, *k*; *Pain and Disability* rats in *lanes* *a*, *b*, *j*; *Pain and Transient Disability* rats in *lanes* *c*, *d*, *l*; sham control rats in *lanes* *m*, *n*, *o*; and behavioural controls in *lanes* *p*, *q*).

Quantitative real time RT-PCR was used to determine relative TH mRNA expression and western blotting was used to determine relative TH protein expression levels in the midbrains of CCI and control rats; these results are presented in Fig. 2. Rats with *Pain and Transient Disability* had increased levels of TH mRNA compared to each of the control groups (F_4,32_ = 4.95, p < 0.01) (Fig. 2a). The Western blots identified TH and beta-actin with single bands of ~60 kDa and ~43 kDa, respectively (Fig. 2c). TH protein levels were calculated with respect to the *housekeeping* protein beta-actin; then the relative expression of TH in CCI and sham injured rats was expressed relative to behavioural controls (Fig. 2c). *Pain and Transient Disability* rats showed a significant increase in TH protein expression compared to both behavioural and sham controls (F_4,31_ = 5.9, p < 0.01), and compared to *Pain alone* rats (F_4,31_ = 5.9, p < 0.05) (Fig. 2b).

### Immunohistochemical analysis

**Fig. 3:**
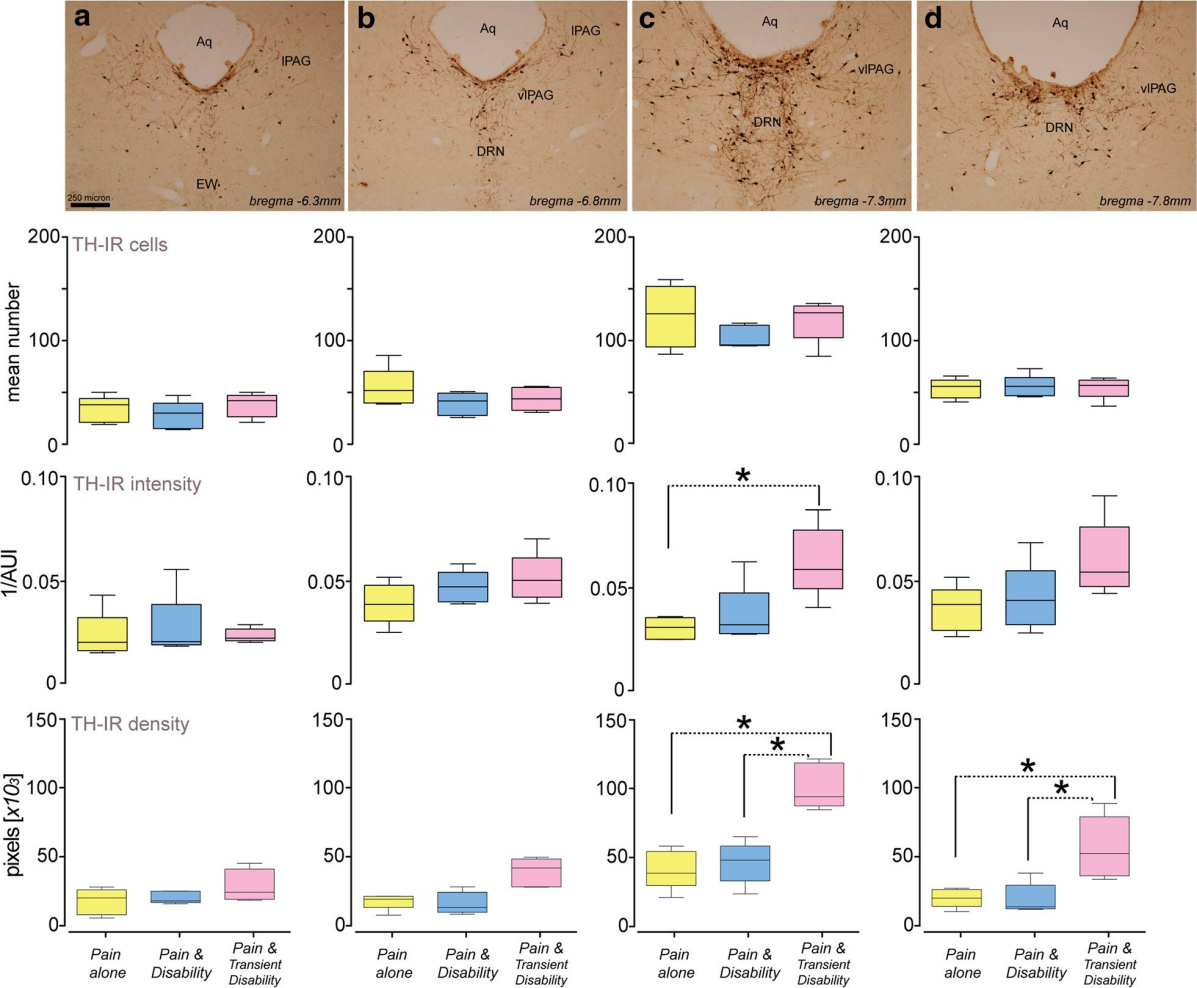
TH-expression in the vPAG of rats with distinct patterns of injury-triggered disability. *Top row* **a**–**d** shows photomicrographs of TH immunoreactivity in the vPAG under brightfield illumination. **a** −6.3 mm caudal to bregma; **b** −6.8 mm caudal to bregma; **c**, −7.3 mm caudal to bregma; and **d** −7.8 mm caudal to bregma. The *scale bar* represent 1.0 mm. Directly below each photomicrograph, *box* and *whisker plots* illustrate (1) the mean number (±SEM) of TH-IR cells; (2) the mean intensity (±SEM) of staining in TH-IR neurons and; (3) the mean density (±SEM) of TH-IR fibers, at each of these rostro-caudal levels of the vPAG in five rats from each of the behaviourally characterized groups. Significant differences between behavioural groups is shown *p < 0.05 (2-way ANOVA, Bonferroni test).

In injured rats, TH-IR cells and fibers were observed in midbrain sections under the light microscope. TH-IR cell profiles were found in the intermediate lateral PAG; the ventrolateral PAG and the DRN (Fig. 3, top row). In all rats evaluated the largest numbers of TH-IR cells were found in the DRN at approximately −7.3 mm from bregma (Fig. 3, second row, column c). However these cell numbers did not differ between groups. TH-IR in the neurons of rats with *Pain and Transient Disability* was significantly greater than that in *Pain alone* rats at −7.3 mm from bregma (F_11,48_ = 5.443, p < 0.05) (Fig. 3, third row, column c). Further, rats with *Pain andTransient Disability* also showed increased densities of TH-IR fibers at 7.3 and 7.8 mm caudal to bregma, compared to both *Pain and Disability* and *Pain alone* rats (F_11,48_ = 19.97, p < 0.001) (Fig. 3, fourth row, columns c, d).

## Discussion

The aims of this study were to identify alterations in tyrosine hydroxylase expression in the ventral PAG 6 days after sciatic nerve CCI, a time point at which we have shown that rats segregate into three distinct behaviourally defined groups, despite the presence of identical sensory disruptions in all rats. These groups are: (1) *Pain and Disability*, a subgroup in which dominance and non-social behaviour in the resident-intruder social interactions test, and sleep-wake cycles are disrupted; (2) *Pain and Transient Disability*, a subgroup in which CCI disrupted dominance and non-social behaviour in the resident-intruder social interactions test are restored to normal levels, but sleep–wake cycles in the light phase are disrupted and; (3) *Pain alone* a subgroup which experience no disruptions in either resident intruder interactions or the sleep–wake cycle. Real-time RT-PCR and western blotting revealed increased TH expression, at both transcription and translation levels in the midbrain of rats with *Pain andTransient Disability* 6 days post-CCI. Systematic evaluation of staining for TH-IR in the midbrain revealed that the number and location of TH-IR cells in the vPAG region did not differ between CCI groups, but consistent with the western blotting data, rats in the *Pain and Transient Disability* group had significantly greater densities of TH-IR fibres and significantly greater intensity of TH immunoreactivity in vlPAG and DRN neurons [i.e., the A10dc and A6 (rostral) groups] than rats in the *Pain alone* and *Pain and Disability* subgroups.

These data provide evidence for a selective increase in tyrosine hydroxylase expression in the constitutive vPAG TH-IR cell populations [A10dc and A6 (rostral) cell groups], and increased TH-IR in vPAG fibres, in rats that show an initial decrease in dominance and increase in non-social behaviours for the first 2–3 days after CCI, coupled with increased wakefulness and decreased slow-wave sleep during the light phase, a pattern we have reported previously [14] which we term *Pain and Transient Disability*. Increased TH-IR in the A10dc cell group likely indicates increased dopamine synthesis and increased TH-IR in the rostral A6 cells likely indicates increased noradrenaline synthesis. Our earlier reports of a selective increase in plasma corticosterone in *Pain and Transient Disability* rats on days 2 and 3 post-CCI [31] may reveal one mechanism by which TH mRNA can be selectively up-regulated in the vPAG of this behavioural subgroup [32]. Further, increased activity in dopaminergic and noradrenergic projections originating in the vPAG may also contribute to these patterns of TH increase reported here [32, 33]. Finally, the increased density of TH-IR in fibres in the vPAG may reflect increased catecholaminergic drive in this area. We consider each of these suggestions in turn.

The A10dc cells also have extensive ascending and largely non-collateralised projections into the ventrolateral preoptic area and perifornical (orexinergic) lateral hypothalamus, midline and intralaminar thalamus, the central nucleus of the amygdala, the bed nucleus of the stria terminalis, the shell of the nucleus accumbens, the septum, the basal forebrain cholinergic nuclei and the medial prefrontal cortex (mPFC). As well, they project within the midbrain-pontine region to the lateral dorsal tegmental nucleus and the locus coeruleus [16, 23, 24, 34]. The importance of these extensive projections in regulating the behavioural responses to environmentally salient stimuli is well known [35]. A role for the A10dc cells in resident intruder interactions is also suggested by observations that dopamine levels in the nucleus accumbens (NAc) and the mPFC increase during resident-intruder interactions and these increases are sustained for up to 20–30 min. Further, daily resident-intruder testing results in an anticipatory increase in dopamine in the NAc prior to the testing period [36, 37]. In rats with *Pain and Transient Disability* an increase in A10dc DA activity in cells which project into the NAc and mPFC may represent an increased drive to engage the intruder in the stereotyped behaviours disrupted by CCI in the early (days 1–3) post-CCI phase. This suggests that for the subgroups of rats in which, CCI disrupts resident-intruder behaviours (i.e., *Pain and Disability* and *Pain and Transient Disability*) there may be differential changes in dopamine responsiveness of the NAc and mPFC neuronal populations which regulate social interactions between the two behavioural subgroups (see for example [38]).

Neurons of the vPAG are pivotal in the regulation of REM sleep and wakefulness. Inhibition of vPAG neurons with microinjections of the GABA-a agonist muscimol significantly increased the duration of REM sleep [39], and selective lesions of DA vlPAG neurons led to increased wakefulness [16, 40]. These vlPAG DA cells project into multiple loci of the sleep regulatory network, including the cholinergic neurons of the basal forebrain, the ventrolateral preoptic region, the orexin neurons of the lateral hypothalamus, the cholinergic pontine laterodorsal tegmental nucleus and the locus coeruleus. Increased TH expression in the A10dc cells may contribute to the disrupted sleep-wake cycle seen during the light phase in the *Pain and Transient Disability* rats, this mechanism appears to be different from that underlying the sleep-wake disturbances in *Pain and Disability* rats in which TH levels remained unchanged. Thus whilst we have provided evidence for changes in dopaminergic activity in the A10dc group in a subset of CCI rats, changes in dopamine receptor expression in the target areas of these neurons following nerve injury requires more systematic evaluation.

The A6 noradrenergic cells found in the vPAG comprise the rostral cell group [17], this group of cells have been shown to be critical in regulating wakefulness and controlling levels of arousal and alertness [41], further they play a role in the regulation and promotion of social interactions [42]. Increased TH in this small group of cells may contribute to the restoration of normal resident intruder social interactions following post CCI days 2–3, as well as regulating wakefulness during the light phase.

The non-somatic TH-IR in the vPAG may be located in either the dendrites of the immunoreactive neurons or in input terminals and fibres *en passant* originating in the catecholaminergic cell groups of the lower brainstem. Catecholaminergic cells, which project to the vPAG include the A1 and C1 cells of the ventrolateral medulla [43]; the A2 and C2 cell groups of the dorsomedial medulla [44] and the A6 cells of the locus coeruleus [45]. Recent evidence indicates that CCI increases noradrenaline synthesis in the locus coeruleus [46, 47]. The extent to which a selective activation of ascending medullary adrenergic and noradrenergic pathways contributes to the pattern of TH-IR in the vPAG of rats with *Pain and Transient Disability* remains to be established experimentally.

## Methods

The approval of the University of Sydney Animal Care and Ethics Committee, following the guidelines of the *NHMRC* “Code for the Care and Use of Animals in Research in Australia”, the NSW Animal Research Act (2007) was given for these experiments, which also adhered to the “Ethical Guidelines for Investigations of Experimental Pain in Conscious Animals” laid down by the International Association for the Study of Pain [28].

### Behavioural testing

Outbred, male Sprague–Dawley rats, 250–350 g at the time of surgery (ARC Perth, WA, Australia) were used in these experiments. They were maintained on a reversed 12/12 light/dark cycle with food and water available ad libitum. Room temperature was maintained at 22 (±1)°C. Behavioural tests were conducted during the dark phase of the circadian cycle.

‘Resident-intruder’ testing was performed as described by Monassi and colleagues [14]. Briefly, rats were individually housed in clear perspex cages for 7 days (the ‘resident’). For the next 6 days, approximately 2 h after lights off, a novel, age and weight matched ‘intruder’ was introduced into the cage and the resulting social interactions were video-recorded for a period of 6 min (pre-CCI). On the next day, the resident rat was given a sciatic nerve CCI (see below), and a day later, the resident intruder testing continued for a further 6 days (post CCI). The behaviours of the resident rats, in response to the intruder were scored, and the durations of four mutually exclusive categories of behaviour (dominance, social, non-social and submissive) were quantified.

On day 6 post-CCI, rats were either decapitated rapidly under CO_2_ narcosis, and the brains removed immediately, snap frozen in liquid nitrogen and stored at −80°C until assayed (N = 52), or they were deeply anaesthetized with sodium pentobarbitone (Nembutal: 120 mg/kg i.p.) and perfused intra-cardially with 0.9% saline (500 ml room temperature) followed by 4% paraformaldehyde in phosphate buffer (pH 7.4, 4°C) (N = 15)

### Behavioural analyses

Resident behaviours in the 6 days following sciatic nerve constriction injury were compared to those in the 3 days prior to injury (i.e. pre-CCI days 3–5). On the basis of these comparisons, rats were categorised into one of three distinct behavioral groups. Rats that showed no differences in their behaviour following CCI were considered *Pain alone* rats; rats with a decrease of at least 30% in the duration of their dominance behaviours in 4 of the 6 days post injury days were classified as *Pain and Disability* rats. The rats that showed an initial reduction in their dominance behaviours for the first 3–4 days but then returned to pre-CCI levels by day 6 were considered *Pain and Transient Disability* rats. The duration of time spent in each category of resident-intruder behaviour for each behavioural group were expressed as means ± (SEM). Comparisons between groups were made with a two-way ANOVA and significant differences between groups were determined post hoc using Fischer’s protected least significance difference (PLSD) test.

### Sciatic nerve CCI

Sciatic nerve chronic constriction injuries were performed, by following exactly the procedure described by Bennett and Xie [29]. Under halothane anaesthesia (2% in oxygen) the right sciatic nerve was exposed by gentle blunt dissection. Four chromic gut ligatures were tied loosely around the sciatic nerve approximately 1 mm apart. The overlying muscles were closed, the skin incision sutured and the wound dusted with topical antibiotic powder (Tricin).

### Control groups

Two groups of control rats were used in these experiments. Sham controls (N = 6) underwent resident-intruder testing as well as sham surgery in which they were placed under halothane anaesthesia (2% in oxygen) and the sciatic nerve was exposed but no CCI was performed. Behavioural controls (N = 24) received daily resident-intruder testing but were not anaesthetised neither did they experience any surgical procedures. Twenty-four behavioural control rats were used to compare with the 26 rats that received CCI and were subsequently processed for RT-PCR and western blots.

Following the last day of resident-intruder testing, rats were either rapidly decapitated [N = 56: (*Pain alone* = *11*; *Pain and Disability* = *10*; *Pain and Transient Disability* = *5*; *Behavioural Control* = *24*; *Sham Control* = *6*)] and the brains removed and stored in −80°C until used, or they were deeply anaesthetised with sodium pentobarbitone (i.p., Nembutal 120 mg/kg) and perfused transcardially with 500 ml of heparinised 0.9% saline at room temperature, followed by fixation with 600 ml of 4% paraformaldehyde in phosphate buffer (pH 7.4; 4°C) [N = 15: (*Pain alone* = *5*; *Pain and Disability* = *5*; *Pain and Transient Disability* = *5*)].

### Real-time RT-PCR

A dorsal midbrain block encompassing the PAG and DRN was isolated from each of the frozen brains. The rostral boundary of this block was at the approximate coronal level of −5.3 mm caudal to bregma and the caudal boundary at approximately −8.8 mm caudal to bregma [30]. The PAG region surrounding the mesencephalic aqueduct was dissected free under a binocular microscope, weighed and total RNA of the isolated tissue was extracted using TRI reagent (Sigma-Aldrich). RNA concentrations were calculated using spectrophotometry (ND-1000 spectrophotometer, NanodropTH, Wilmington, DE, USA) and RNA integrity was verified using the 2100 Bioanalyser (Agilent Technologies™, Santa Clara, CA, USA). 1 μg of total RNA was used for cDNA synthesis (Tetro Reverse transcriptase, Bioline, Australia). Relative expression levels of TH mRNA were determined using TaqMan^®^ FAM probe with TAMRA as the quencher dye (Rn00561369_m1). GAPDH was used simultaneously as a housekeeping gene using a VIC probe with MGB as the quencher dye (product Number 4352338E).

### Western blotting

Protein was isolated from the organic phase in the TRI reagent protocol. Total protein concentrations for each midbrain block were estimated using the Direct Detect™ Spectrometer (Millipore, Bedford, MA, USA). 15 μg of total protein was then separated on 12% Criterion TGX stain-Free gels (Bio-Rad) and transferred to PVDF membranes using the trans-Blot^®^ Turbo Transfer packs (Bio-Rad). Membranes were blocked in skim milk powder (5%) and incubated overnight at 4°C with a mouse monoclonal anti-TH antibody (1:10,000, ImmunoStar), followed by incubation with HRP-conjugated goat anti-mouse IgG (1:5,000, Santa Cruz). TH was visualised using the Immobilon Western chemiluminescence HRP substrate (Millipore, Bedford, MA, USA) on a ChemiDoc MP imaging system (Bio-Rad). Bands were quantified using Image Lab™ software.

Membranes were then incubated in rabbit polyclonal anti-beta actin antibody (1:1,000, Santa Cruz) followed by HRP-conjugated goat anti-rabbit IgG (1:5,000, Santa Cruz). The labeled beta actin was visualised and quantified as described above.

### Immunohistochemistry

Following transcardial perfusion with fixative, the brains of each rat were removed and post-fixed overnight in 4% paraformaldehyde (4°C) and then placed in 30% sucrose in 0.1 M phosphate saline buffer (PBS) at 4°C until the brains sank. A one in ten series of 20 μm sections were cut through from −5.8 to −8.8 mm from bregma using a freezing microtome (Leica, Germany). All sections were treated identically and were reacted at each stage, for exactly the same time. Sections were incubated in 3% H_2_O_2_ to block endogenous peroxidase activity, blocked in 10% normal horse serum (NHS) and 0.3% triton-X in PBS and then incubated in a mouse monoclonal anti-TH antibody (1:10,000, ImmunoStar) at 4° overnight. Sections were washed in PBS then incubation in biotinylated donkey anti-mouse IgG (1:500; Jackson Immunochemicals, West Grove, PA, USA) for 2 h. Following a wash in PBS, the sections were then incubated in ExtrAvidin peroxidase (1:1,000; Sigma-Aldrich, St. Louis, MO, USA) for 2 h. The bound antibody complex was visualised using DAB (0.05%) as the chromagen. Sections were mounted onto gelatinized slides; allowed to dry overnight; dehydrated through graded alcohols and then coverslipped with DPX mounting medium.

### Image analysis

Analysis of tyrosine hydroxylase immunoreactivity (TH-IR) was done under the light microscope (Olympus BX51). In each rat (N = 15), the number of TH-IR neurons was counted on coronal sections corresponding to atlas sections from the atlas of Paxinos and Watson [30] at 6.3, 6.8, 7.3 and 7.8 mm caudal to the skull marking bregma. The staining intensity of the TH-IR neurons and the density of TH-IR fibers on each of these sections were calculated using Metamorph^®^ (Molecular Devices, Inc). A digital image of each section at 100× magnification was captured under identical illumination and resolution using a light microscope (Olympus BX) and digital camera (Olympus DP70). Each image was opened in the Metamorph^®^ software and colour thresholding was used to selectively highlight TH-IR neuronal cell bodies. Integrated morphometry analysis was then used to determine the average intensity of the TH-IR cell population, normalised against background staining. Following this, in a randomly selected section the color thresholding function was used to identify the threshold range of non-somatic i.e., fibre and dendrite TH-IR. This threshold range was then applied to all sections to quantify the density of non-somatic TH-IR.

Comparisons of TH-IR cell numbers, TH-IR intensity in neurons and TH-IR density in non-somatic structures were made between the behavioural groups (*Pain alone*, *Pain and Disability and Pain and Transient Disability*) across the rostro-caudal levels (*6.3*, *6.8*, *7.3*, *7.8* *mm caudal to bregma*) using a one-way ANOVA, and when significance was found, the specific locations of the differences were determined by interrogating the data using the Bonferroni post hoc test. Statistical significance was set as *p* < 0.05.

## Abbreviations

DA: dopamine
DRN: dorsal raphe nucleus
CCI: chronic constriction injury
lPAG: lateral periaqueductal grey column
mPFC: medial prefrontal cortex
NAc: nucleus accumbens
PAG: periaqueductal grey
SWS: slow wave sleep
RT-PCR: reverse transcription polymerase chain reaction
TH: tyrosine hydroxylase
TH-IR: tyrosine hydroxylase immunoreactive
vPAG: ventral periaqueductal grey region
VTA: ventral tegmental area
